# Combining affinity proteomics and network context to identify new phosphatase substrates and adapters in growth pathways

**DOI:** 10.3389/fgene.2014.00115

**Published:** 2014-05-07

**Authors:** Francesca Sacco, Karsten Boldt, Alberto Calderone, Simona Panni, Serena Paoluzi, Luisa Castagnoli, Marius Ueffing, Gianni Cesareni

**Affiliations:** ^1^Department of Biology, University of Rome Tor VergataRome, Italy; ^2^Division of Experimental Ophthalmology, Centre for Ophthalmology, Institute for Ophthalmic Research, University of TuebingenTuebingen, Germany; ^3^Department DiBEST, University of CalabriaRende, Italy; ^4^Research Unit for Protein Science, Helmholtz Zentrum MünchenNeuherberg, Germany; ^5^Istituto Ricovero e Cura a Carattere Scientifico, Fondazione Santa LuciaRome, Italy

**Keywords:** phosphatase, signal transduction, systems biology, cell biology, protein protein interaction

## Abstract

Protein phosphorylation homoeostasis is tightly controlled and pathological conditions are caused by subtle alterations of the cell phosphorylation profile. Altered levels of kinase activities have already been associated to specific diseases. Less is known about the impact of phosphatases, the enzymes that down-regulate phosphorylation by removing the phosphate groups. This is partly due to our poor understanding of the phosphatase-substrate network. Much of phosphatase substrate specificity is not based on intrinsic enzyme specificity with the catalytic pocket recognizing the sequence/structure context of the phosphorylated residue. In addition many phosphatase catalytic subunits do not form a stable complex with their substrates. This makes the inference and validation of phosphatase substrates a non-trivial task. Here, we present a novel approach that builds on the observation that much of phosphatase substrate selection is based on the network of physical interactions linking the phosphatase to the substrate. We first used affinity proteomics coupled to quantitative mass spectrometry to saturate the interactome of eight phosphatases whose down regulations was shown to affect the activation of the RAS-PI3K pathway. By integrating information from functional siRNA with protein interaction information, we develop a strategy that aims at inferring phosphatase physiological substrates. Graph analysis is used to identify protein scaffolds that may link the catalytic subunits to their substrates. By this approach we rediscover several previously described phosphatase substrate interactions and characterize two new protein scaffolds that promote the dephosphorylation of PTPN11 and ERK by DUSP18 and DUSP26, respectively.

## Introduction

Protein phosphorylation is a common post-translational modification governing signal propagation (Mann and Jensen, 2003). The concerted activity of kinases and phosphatases modulate protein phosphorylation levels and control key physiological processes, such as migration, proliferation, inflammation, and apoptosis (Graves and Krebs, 1999; Manning et al., 2002a,b). Till recently protein phosphatases have been considered uninteresting housekeeping enzymes and have received less attention compared to kinases (Bardelli and Velculescu, 2005). However, evidence accumulated over the past decades have indicated that this enzyme class plays an important regulatory role and that the deregulation of the concentration or activity of specific phosphatases correlate with a variety of human disorders (Wera and Hemmings, 1995; Tonks, 2006). Notably, approximately 40% of protein phosphatases are implicated in tumor development, highlighting the central role of this enzyme group in growth regulation and identifying some members of this enzyme class as promising therapeutic targets (Julien et al., 2011; Liberti et al., 2013). One of the problems in the characterization, on a large scale, of the functional role of members of the phosphatase family is the lack of a simple, robust, method to identify physiologically relevant substrates. Many phosphatases have low intrinsic enzymatic specificity and are able to de-phosphorylate many substrates non-specifically *in vitro* (Tremblay, 2009). Alternative methods such as the use of trapping mutants (Blanchetot et al., 2005) are often used, but the identification of direct phosphatase substrates still remains a challenge.

In order to characterize new modulators of some key cancer associated pathways and to identify their direct targets, we have recently proposed a novel strategy based on a phosphatase high content siRNA screening combined with modeling and simulation. This approach enabled the identification of 62 phosphatase catalytic or regulatory subunits whose down-regulation affects one or more of five readouts linked to cell proliferation: ERK, p38, and NFkB activation, rpS6 phosphorylation and autophagy (Sacco et al., 2012a). However, this approach was not designed to identify the direct phosphatase substrates, responsible for the phenotypic effect.

**Figure 1:**
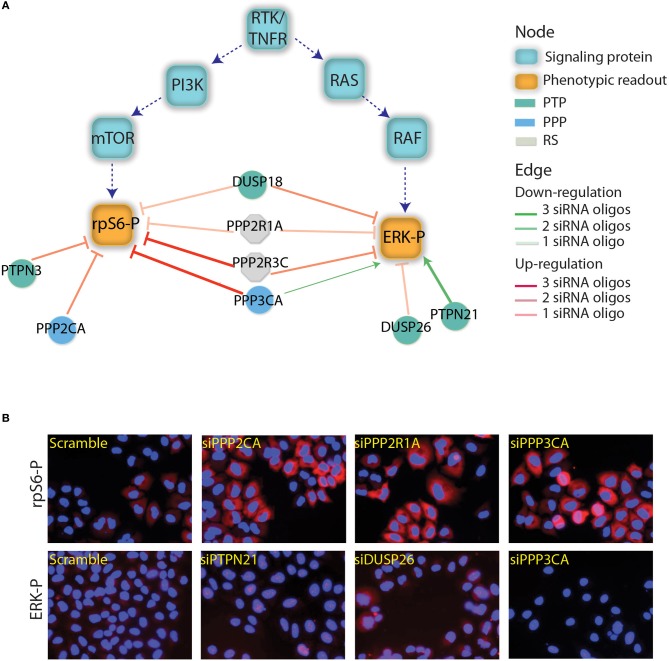
**Phosphatases affecting the RAS-PI3K pathway. (A)** The results depicted in this figure are a subset of the results reported by Sacco et al. (2012a). The squares represents the main proteins that are involved in the PI3K and RTK/RAS signaling cascades. Green squares are the phosphorylated proteins that have been used as readouts in the screening. The phosphatase whose down regulation was shown to affect the readouts are represented as circles and are linked to readouts with red or green edges depending on whether their down-regulation negatively or positively affected the readouts. The intensity of the edge red or green color represents the trust in the reported interaction (number of concordant siRNA). The color of the phosphatase nodes is mapped to the phosphatase family according to the figure legend. **(B)** As previously described by Sacco et al., after phosphatase down-regulation the activation level of ERK and rpS6 was analyzed by automated immunofluorescence microscopy. Here, we report the ERK and rpS6 phosphorylation level after the down-regulation of some phosphatases hits.

Here we delineate a strategy to identify protein scaffolds that may contribute to substrate recognition specificity by bridging the phosphatases to their targets. To develop this strategy we focused on eight phosphatase subunits whose down-regulation was found to affect ERK and/or RPS6 phosphorylation and are therefore modulators of the RAS-PI3K pathway (Figure 1). To identify new phosphatase substrates involved in the control of the RAS-PI3K pathway we first built a protein interaction network (PPI) by combining information extracted from protein interaction databases and results from new affinity purification experiments. This analysis confirmed that the identified phosphatase interactors often act as molecular bridges linking enzymes to substrates. In addition we independently validated a subset of these predictions

## Results

### The phosphatase interactome

**Figure 2:**
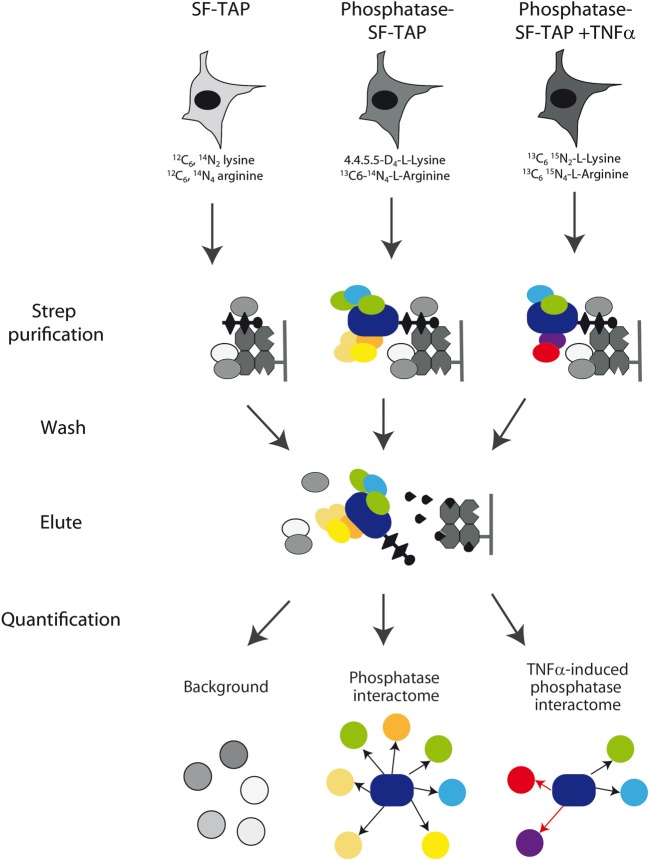
**Experimental strategy**. Schematic overview of the experimental strategy applied to analyze the phosphatase interactome. Phosphatase transfected HeLa cells with or without TNFα were grown in media containing heavy isotope labeled lysine and arginine amino acids, while the control cells were grown in a medium containing natural amino acids. After lysis, phosphatases, and regulatory subunits were affinity purified by Strep purification and analyzed by mass spectrometry as described in Materials and Methods section. Phosphatase interactors were identified and quantified by their significant enrichment compared to the control and TNFa-induced alterations were quantified.

**Figure 3:**
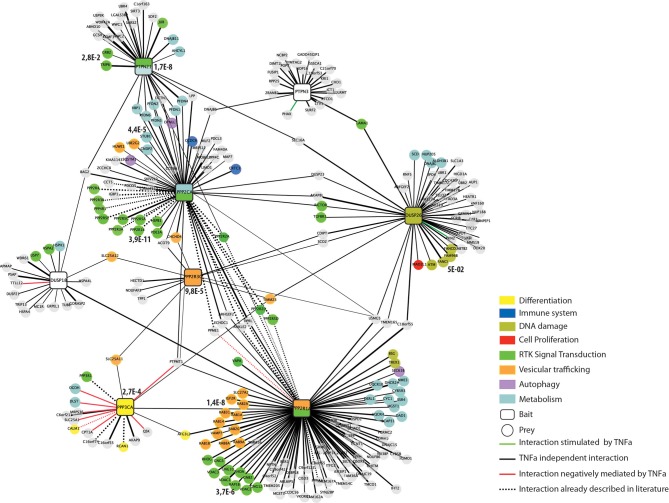
**The human phosphatase interactome**. Phosphatases (squares) are linked to the experimentally identified interactors (circles) by edges. Edges are colored according to the functional relationships between the nodes they connect: interactions positively regulated by TNFα are in green; interactions negatively regulated by TNFα are red and TNFα independent interactions in black. Dashed lines represent interactions that have already been reported in the literature. Interactors are colored according to their functional association as revealed by our Kegg-Pathways enrichment analysis performed by the DAVID software. The phosphatase nodes are labeled according to the Kegg pathways that was significantly overrepresented in the phosphatase interactors and substrates (*p*-value < 0.005).

We have used the results of the siRNA screening (Sacco et al., 2012a) to select eight phosphatases that modulate the activity of the RAS-PI3K pathway. The phosphatase catalytic or regulatory subunits were cloned in frame C-terminal to an SF-TAP cassette and transiently transfected in HeLa cells. These constructs direct the synthesis of four tyrosine phosphatases (PTPN21, PTPN3, DUSP18, and DUSP26), three components of the PP2A holoenzyme (the PPP2R3C regulatory subunit, the PPP2R1A scaffold subunit and the PPP2CA catalytic subunit) and the PPP3CA (calcineurin) serine/threonine phosphatase. As negative control, HeLa cells were transiently transfected with the empty vector SF-TAP. Since in our siRNA screening phosphatases controlling the activity of the RAS-PI3K pathway were identified in HeLa cells stimulated with TNFα for 10 min, we decided to perform the affinity purification experiments in the same experimental condition. Thus, phosphatase transfected cells were stimulated with TNFα for 10 min or left untreated. While the control cells were grown in a medium containing natural amino acids, phosphatase transfected cells with or without TNFα were, respectively, grown in media containing isotopically labeled lysine and arginine amino acids (SILAC) (Ong et al., 2002). After lysis, phosphatases, and regulatory subunits were affinity purified and analyzed by mass spectrometry (Table S1), as described in Materials and Methods (Figure 2). Contaminants binding to these baits were identified by their equal abundance in both (Table S3), the affinity-purified phosphatase sample and the negative control (Meixner et al., 2011), whereas true co-purified interactors to a given phosphatase were identified by selective enrichment of their peptides (Table S2). Only those that were significantly enriched in our samples were considered for further analysis, as described in Materials and Methods. As shown in Figure 3, this strategy resulted in a highly connected interaction network. Approximately 10% of the identified interactions have already been reported in the literature. Indeed we were able to recapitulate most of the interactions occurring between the catalytic and the scaffold subunits of the PP2 holoenzyme, which, as expected, share a significant number of common interactors, many of which are regulatory subunits. These observations taken together with the validation by coimmunoprecipitation assays of some of the newly identified phosphatase interactions (Figure S1) confirm the reliability of our experimental approach. In addition in Figure S1, we demonstrated that our affinity purification experiments enabled the identification of new phosphatase interactors (the dynein protein, DLC1 and the serine threonine kinase, ATM) already involved in the regulation of the autophagy process. With the exception of the PPP2CA-DLC1 binding, both DUSP26-ATM and PTPN21-DLC1 associations are decreased upon an autophagic stimulus (starvation), suggesting that these interactions may have a regulatory role in the autophagy process.

Since the affinity purification was also carried out with or without incubation with TNFα we can also provide dynamically regulated interactions in response to TNFα treatment. As shown in Figure 3, a few interactions are positively (green edge) or negatively (red edge) regulated by TNFα incubation. The vast majority of the co-purified ligands, however, are TNFα independent (black edges).

### Guilt by association

Next we used the phosphatase interactome derived from the *in vivo* pull down experiment to ask whether the phosphatase interaction network could provide hints toward specific pathways that are affected by phosphatase activity. To this aim, we performed a KEGG- pathway enrichment analysis of the ligands of each of the phosphatases, by using the Functional Annotation Tool, David (Huang da et al., 2009). The two phosphatases PTPN21 and PPP2CA and the PP2 scaffold subunit PPP2R1A were significantly associated to RTK signaling (Figure 3), in agreement with their established involvement in the modulation of EGF signaling by controlling the SRC and S6K kinases, respectively (Cardone et al., 2004; Carlucci et al., 2010; Hahn et al., 2010). In addition our enrichment analysis reveals a statistically significant association of PPP3CA with cell differentiation signaling. This result is consistent with the report by Kao et al. that the differentiation of Schwann cells requires the activity of the PPP3CA phosphatase (Kao et al., 2009). Similarly, we found that DUSP26 is significantly correlated with the DNA damage response. This conclusion is in accordance with the observations that DUSP26 inhibits the p53 tumor suppressor function, by suppressing doxorubicin-induced apoptosis in human neuroblastoma cells (Shang et al., 2010). On the other hand, our experimental strategy led to the identification of new biological processes that are controlled by these protein phosphatases (e.g., vesicular trafficking and cell metabolism). As shown in Figure 3, the interactors of both DUSP18 and PTPN3 were not significantly associated to any specific pathway.

**Figure 4:**
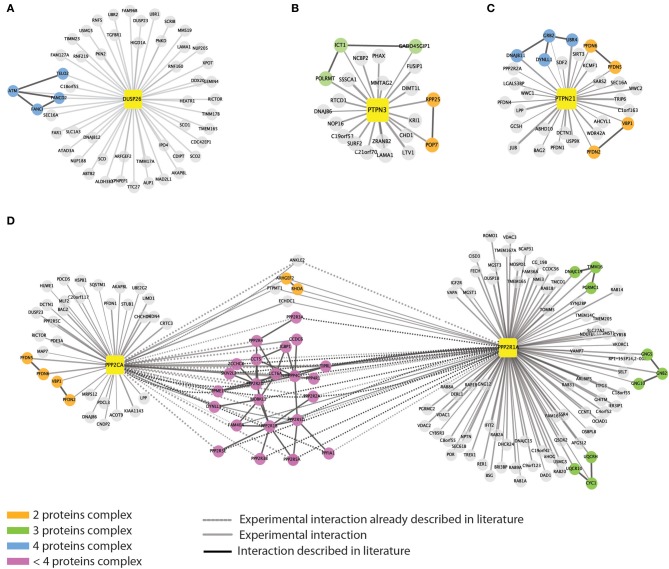
**Evidence for detection of multi-protein complexes**. We queried the mentha protein interaction database to look for direct interactions among the phosphatase interactors. For five out of the eight baits we were able to retrieve information on direct interactions between the affinity purified interactors. Phosphatases are represented as yellow squares. DUSP26 **(A)**, PTPN3 **(B)**, PTPN21 **(C)**, and PPP2CA **(D)** are linked to their substrates by different edges. As in Figure 1, continuous and dashed gray lines represent experimental and literature supported interactions, respectively. Black edges indicate direct interactions occurring among the phosphatase interactors. The color of the nodes is mapped according to the size of the putative complexes formed by the proteins according to the figure legend.

Next we looked for evidence that the proteins that copurified with each phosphatase may form complexes. To this end, we queried the *mentha* database (Calderone et al., 2013), and looked for evidence of interactions between ligands of each bait phosphatase. The interactors of five of the eight phosphatases are linked by direct interactions. As illustrated in Figure 4A, we found that DUSP26 copurifies with the serine/threonine kinase ATM, which, in response to genotoxic stress, phosphorylates the two Fanconi proteins FANCI and FANCD2, triggering the S-phase checkpoint activation (Taniguchi et al., 2002). A third DUSP26 ligand, TELO2, which is a member, together with TTI1 and TTI2, of the TTT complex (Hurov et al., 2010) also interacts with ATM.

PTPN21, on the other hand, interacts with the scaffold protein GRB2, which associates with DNAJB11, DYNLL1, and UBR, suggesting that some of the identified interactors may copurify by indirect interactions (Figure 4C). Similarly we found that PTPN3 interact with the mitochondrial ribosomal subunit ICT1 that binds GADD45GIP1 and POLRMT1 proteins (Figure 4B).

As expected, the catalytic and scaffold subunit of PP2A share many interactors, confirming that such heterodimer forms different protein complexes that act on distinct substrates, by recruiting multiple regulatory subunits (Figure 4D).

### PTPN21 associates with the SH3 domain of GRB2

Among all phosphatase-interaction partners, we focused on the newly discovered interaction between the scaffold protein GRB2 and the tyrosine phosphatase PTPN21, both partners mapping to the RAS-PI3K signaling pathway. Cardone et al. reported that PTPN21 is recruited to mitochondria by binding the scaffold protein AKAP121 and that this interaction is essential for the phosphatase dependent dephosphorylation of the inhibitory tyrosine 527 of the SRC kinase (Cardone et al., 2004; Carlucci et al., 2010).

**Figure 5:**
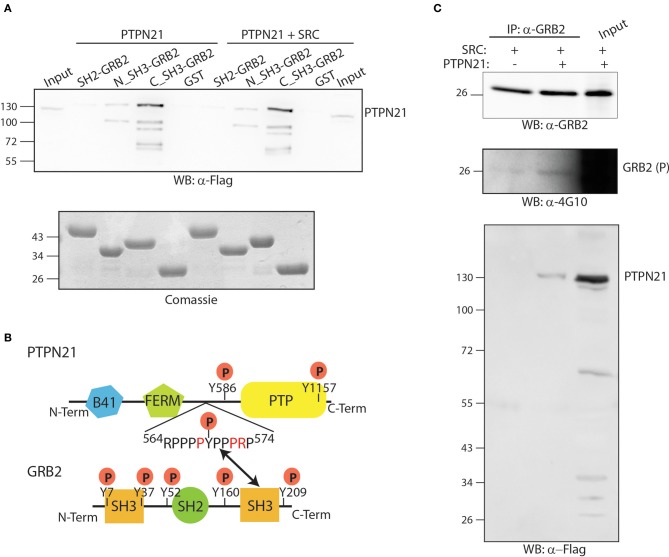
**The PTPN21 phosphatase physically associates with the C-terminal SH3 domain of GRB2. (A)** GST fusion proteins of the N-terminal, C-terminal SH3 domain as well as the SH2 domain of GRB2 were incubated with a protein extract of HeLa cells transiently transfected with Flag-PTPN21 in presence or in absence of constitutively activated Y527F SRC kinase. Affinity-purified SH2 and SH3 domains ligands were separated by SDS-PAGE and transferred onto cellulose membranes. The blots were probed with anti-Flag (WB: α-4G10) and anti-phosphotyrosine (WB: α-4G10) antibodies. The cell lysate (input) and the sample affinity-purified with the GST protein (GST) were used as controls. **(B)** A schematic representation of the modular domain structure of GRB2 and PTPN21 proteins is represented. According to our data, the SH3 binding motif (^564^RPPPPYPPPRP^574^) of PTPN21 binds the GRB2 C-terminal SH3 domain. **(C)** HeLa cells were transiently co-transfected with Flag-PTPN21 and with constitutively activated Y527F SRC kinase expression plasmids. After cell lysis, whole protein extracts were immunoprecipitated with anti-GRB2 antibody. The membrane was probed with anti-GRB2 (WB: α-GRB2), anti-Flag (WB: α-Flag) and anti-phospho tyrosine (WB: α-4G10) antibodies.

GRB2 is an essential adapter protein consisting of two SH3 domains flanking one central SH2 domain. The affinity purification assay results suggest that the GRB2-PTPN21 interaction is not likely to occur in a phosphorylation dependent manner, since it is not modulated by TNFα (Figure 3). However, phosphoproteomics of both cancer and embryonic stem cells revealed that PTPN21 contains multiple tyrosine phosphorylated residues (Rikova et al., 2007; Guo et al., 2008; Brill et al., 2009). In order to map the GRB2-PTPN21 interaction to a specific GRB2 domain and assess whether such binding occurs in a phosphorylation dependent manner, the SH2 domain of GRB2 as well as its two SH3 domains were purified as GST fusion proteins and incubated with whole protein extracts co-transfected with Flag-PTPN21 in presence or in absence of a constitutively active SRC kinase mutant (Y527F). As shown in Figure 5A, PTPN21 strongly binds the C-terminal SH3 domain of GRB2 and to a lesser extent the N-term SH3 domain, independently from SRC. On the other hand, the GRB2 SH2 domain does not interact with PTPN21. The analysis of PTPN21 protein sequence reveals that it contains a SH3 binding motif (^564^RPPPPYPPPRP^574^), whose sequence matches the GRB2 binding specificity described by Carducci et al. (2012). These results support the existence of a PTPN21-GRB2 complex that is phosphorylation independent and likely occurs between the carboxy-terminal SH3 domain of GRB2 and PTPN21 (Figure 5B).

Next we asked whether the formation of the PTPN21 GRB2 complex promotes the dephosphorylation of GRB2. For this purpose, GRB2 tyrosine phosphorylation was induced by transfecting HeLa cells with the constitutively active SRC kinase mutant (Y527F) in presence or in absence of Flag-PTPN21. As shown in Figure 5C, after cell lysis and immunoprecipitation with anti-GRB2, PTPN21 was found to associate with GRB2. However, when PTPN21 is overexpressed, GRB2 phosphorylation, if anything, seems to be slightly increased, as revealed by probing the GRB2 protein with an anti-phospho tyrosine antibody (Figure 5C). Thus, GRB2 is not a substrate of PTPN21 but may play a role in targeting PTPN21 to different substrates.

### A strategy to identify new phosphatase substrates in growth pathways

**Figure 6:**
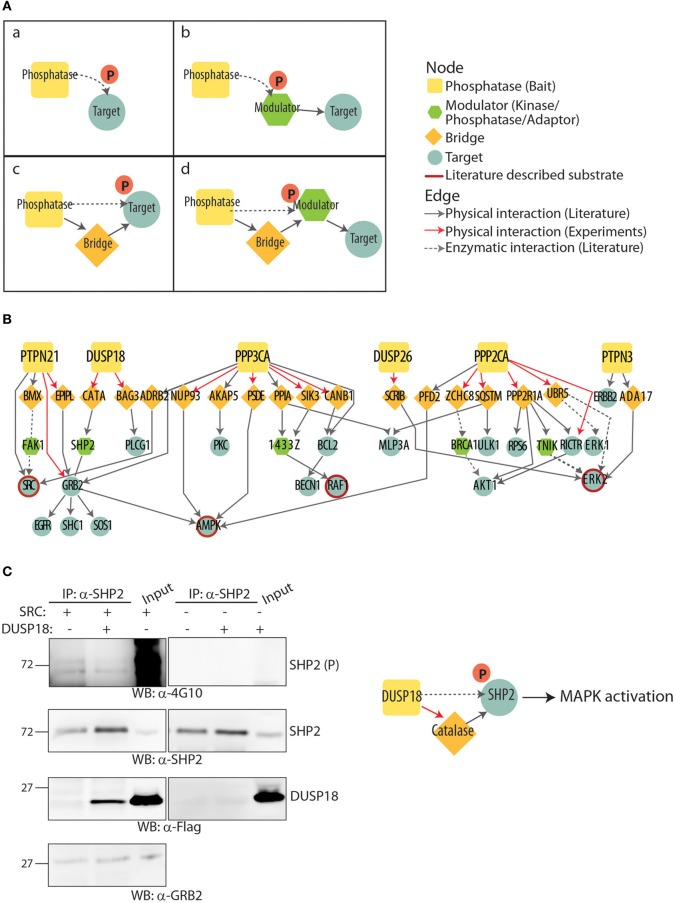
**Inferring new phosphatase substrates. (A)** Schematic representation of the multiple paths going from phosphatase to substrates. **(B)** The multiple paths going from phosphatases to substrates are represented as a graph. Nodes have different shapes according to their functional role: phosphatases are indicated as squares, bridge proteins as diamonds, modulators as hexagons and inferred substrate as circles. The red border outlines phosphatase substrates that have been already reported in literature. Solid black and red lines indicate physical interaction literature and experimentally supported, respectively, while black dashed line represent enzymatic interaction already described in literature. **(C)** HeLa cells were transiently co-transfected with expression plasmids expressing Flag-SHP2 and a constitutively active mutant of the SRC kinase (Y527F) expression plasmids. After cell lysis, whole protein extracts were immunoprecipitated with anti-SHP2 antibody. The beads were washed with lysis buffer, and the immunoprecipitation (IP) was revealed with anti-SHP2 (WB: α-SHP2), anti-GRB2 (WB: α-GRB2), anti-Flag (WB: α-Flag) and anti-phospho tyrosine (WB: α-4G10) antibodies. GRB2 which is an established ligand of SHP2 was used as a positive control.

Having obtained a high coverage interactome of the eight phosphatases that affect the RAS-PI3K pathway we used it to develop a general strategy that could infer the direct target of these phosphatases. Phosphatase-substrate interaction is weak and transient, thus it is unlikely that substrates can be identified by co-immunoprecipitation. In fact none of the interactors identified in the affinity purification experiments are among the validated substrates annotated in the HUPHO and DEPOD databases (Li et al., 2013; Liberti et al., 2013). It has been reported that much of phosphatase substrate specificity, localization and activity is modulated by the interaction with scaffold/regulatory proteins that target them to specific locations (Roy and Cyert, 2009; Sacco et al., 2012b). We hypothesized that some of the interactors identified by our approach act as molecular bridges linking phosphatase to substrates participating in the RAS-PI3K pathway. For this reason, we made use of the PPI network downloaded from the *mentha* database (Calderone et al., 2013) to link phosphatase interactors to putative substrates in the RAS-PI3K pathway (Figure 6A).

The strategy that we used is based on the following steps:

1. Draw a literature derived directed network of the RAS-PI3K pathway and identify the participating proteins as putative targets of the “modulator phosphatases” (Supplementary Material, Table S4).
2. Combine the phosphatase interactors identified in the affinity purification MS experiment with the ones already described in the literature and reported in the *mentha* database (red and black edges, respectively, in Figure 6B).
3. Define paths in the protein interaction graph that connect each phosphatase to the proteins participating in a given pathway (here RAS-PI3K signaling).

By this strategy, each interactor was linked to RAS-PI3K signaling proteins and a by a large number of possible paths. The resulting complex graph was filtered according to the following rules (illustrated in Figure 6A):

1. Longer paths are filtered out. Only paths connecting tyrosine phosphatases to protein members of the growth network with up to two “binding steps” are considered. For phosphatases subunits that form holoenzymes with regulatory subunits such as PP2A and PPP3CA we allowed three binding steps.
2. The inferred substrate in the RAS-PI3K pathway has to be a kinase, a phosphatase or a scaffold protein whose dephosphorylation controls either enzyme activity or the molecular association with other regulatory proteins.
3. The inferred substrate has to contain phosphorylation sites with defined functional roles as annotated in the Phosphosite database (Hornbeck et al., 2012).
4. The phosphorylation sites of the inferred substrates have to be compatible with the nature of the phosphatase (Tyrosine phosphatase can only dephosphorylate tyrosine residues, ect).
5. The effect of the phosphatase induced dephosphorylation of the inferred substrate has to explain the phenotypic effect observed upon phosphatase down-regulation in the functional screenings (Sacco et al., 2012a).

The result of this approach (Table S5) is illustrated in the filtered graph in Figure 6B. Remarkably, our strategy was validated by the recovery of phosphatase substrates already reported in the literature. For instance, Duan and Cobb already demonstrated that PPP3CA induces the MAPK activation by dephosphorylating Thr401 in RAF1 (Duan and Cobb, 2010). In addition the inhibitory effect of PTPN3 on ERK phosphorylation was already reported by Han et al. (2000). Interestingly, both PPP3CA and PPP2CA phosphatases have been already described to be negative modulators of autophagy (Magnaudeix et al., 2013; He et al., 2014). Our approach enabled the identification of a new potential molecular mechanism that these two phosphatases may control to modulate autophagy. SIK3 and SQSTM proteins have been identified by our affinity purification experiment as two novel interactors of PPP3CA and PPP2CA, respectively. In our approach, we propose that SIK3 and SQSTM proteins act as bridge to connect PPP3CA and PPP2CA phosphatases to the autophagy marker MLP3A (LC3A). This observation suggests that our approach can be used to propose new potential molecular mechanisms linking a phosphatase to an established biological process. This graph links phosphatase to putative adapter and to putative substrates. In principle depending on the available information one can use it (1) to infer new substrates starting from a consolidated PPI or (2) to validate molecular bridges that target a phosphatase to an established substrate. In the two following paragraphs we will demonstrate these strategies in two specific cases.

### SHP2 can be dephosphorylated by DUSP18

DUSP18 was shown by our screening to negatively regulate the RAS pathway. The graph in Figure 6B indicates that the regulatory protein that is closest to DUSP18 in the RAS pathway is SHP2 and that DUSP18 and SHP2 are connected by catalase. Indeed it has been shown that the SH2 domains of SHP2 bind tyrosine phosphorylated catalase (Yano et al., 2004), and catalase was recovered as a DUSP18 interactor in our approach. We can therefore picture catalase acting as a bridge linking the phosphatase to its putative target. To test this hypothesis, HeLa cells were transiently co-transfected with Y527F SRC kinase, to enhance phosphorylation, and Flag-DUSP18. As shown in Figure 6C, after cell lysis and endogenous immunoprecipitation with anti-SHP2, DUSP18 was found to associate with SHP2 only in SRC transfected cells. This data is compatible with the model whereby the SH2 domains of SHP2 bind tyrosine phosphorylated catalase which in turn binds to DUSP18. In addition the over-expression of DUSP18 induces SHP2 dephosphorylation, without affecting its association with GRB2. Since it has been shown that the C-terminal tyrosine residues of SHP2 bind GRB2, this result suggests that DUSP18 likely dephosphorylates the Tyr62 and Tyr63 residues. Although the biological relevance of the inferred dephosphorylation needs to be proven in more physiological conditions, this result shows that DUSP18 has the potential to dephosphorylate SHP2 as inferred by our approach.

### SCRIB acts as a bridge to target DUSP26 to ERK

Knock down of DUSP26 by siRNA negatively affects the activation of ERK (Sacco et al., 2012a). This is in agreement with the ability of DUSP26 to inhibit cell proliferation in epithelial cell lines (Hu and Mivechi, 2006; Patterson et al., 2010). Consistent with a role as tumor suppressor, DUSP26 is down-regulated, in several human cancer cell lines, as well as in some primary tumors (Tanuma et al., 2009; Patterson et al., 2010). However, DUSP26 is not able to directly bind ERK to dephosphorylate it (Hu and Mivechi, 2006; Patterson et al., 2010) suggesting the existence of a molecular bridge The heat shock transcription factor Hsf4b, a substrate of ERK, was proposed as a possible bridge to link DUSP26 to ERK (Hu and Mivechi, 2006). Similarly, more recently, the adenylate kinase 2 was proposed to be a bridge that directs DUSP26 to dephosphorylate FADD (Kim et al., 2014).

**Figure 7:**
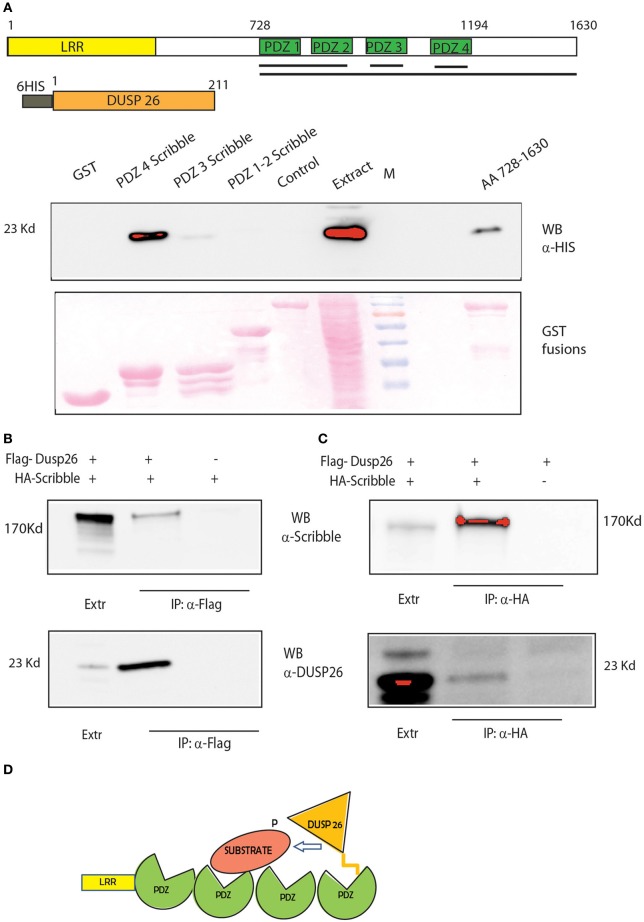
**DUSP 26 interacts with the adapter protein SCRIB. (A)** GST Pull Down of full-length DUSP26 by GST constructs fused to PDZ 1-2, PDZ3, PDZ4, or all the four PDZ domains (aa 728-1630) of SCRIB fused to GST. Black Lines under the schematic representation of the domain structure of SCRIB indicate the protein regions that were fused to GST. Two controls were added: GST alone and a PDZ containing protein of similar length (PDZK1, lane “control”). **(B,C)** Co-immunoprecipitation of SCRIB and DUSP26. H1299 cells were transfected with HA-SCRIB, Flag-DUSP26 or both plasmids as indicated. Lysates were immunoprecipitated with anti-Flag **(B)** or with anti-HA **(C)** and detection was performed with anti-DUSP26 or anti-SCRIB antibodies. **(D)** Cartoon picturing the proposed role of SCRIB as adapter protein to mediate DUSP26 dephosphorylation specificity.

Our approach identified SCRIB as a potential bridge that would modulate the de-phosphorylation of ERK by DUSP26. SCRIB is an adapter protein that was recently suggested to down-regulate ERK by binding and activating the phosphatase PP1 gamma (Nagasaka et al., 2013). We propose here that SCRIB may also promote the de-phosphorylation of ERK by DUSP26. SCRIB directly binds to ERK through two KIM motifs and regulates its activation and nuclear translocation (Nagasaka et al., 2010). The protein contains four PDZ domains (Figure 6A). The C-terminus region of DUSP26 contains an atypical motif for PDZ binding L-D/E-Φ, where Φ is a hydrophobic residue (Tonikian et al., 2008). Thus, we asked whether the binding of SCRIB to DUSP26, as identified in our affinity purification experiment, could be mediated by any of the SCRIB PDZ domains. To this end we performed a GST pull down experiments by affinity purifying extracts of *E. coli* cells expressing HIS-tagged DUSP26 with GST fusion of SCRIB PDZ domains (Figure 7A). Only the fourth PDZ domain of SCRIB was able to bind DUSP26. The binding was confirmed by co-immunoprecipitation assay, after cotransfecting HA-SCRIB and Flag-DUSP26 in H1299 cells. As shown in Figure 7B, co-immunoprecipitated SCRIB was detected by western blotting with an anti-SCRIB antibody. Similarly SCRIB was immunoprecipitated with anti-HA antibody and the presence of DUSP26 was revealed by western blotting with anti-DUSP26 antibody Figure 7C. These data suggest that SCRIB could direct the phosphatase activity of DUSP26 toward ERK as suggested in the cartoon in Figure 7D.

## Discussion

Although protein phosphorylation has been considered as a key post-translational mechanism controlling a variety of physiological processes and a number of reports have contributed to describe the phosphatase interaction network, a comprehensive characterization of phosphatase substrates is still missing (Goudreault et al., 2009; Breitkreutz et al., 2010; Skarra et al., 2011; Couzens et al., 2013). Recently we have reported an unbiased siRNA screening aimed at identifying phosphatases controlling key growth pathways in cancer cells (Sacco et al., 2012a). Combining the siRNA screening results with modeling techniques, we were able to map phosphatases on specific nodes of the growth signaling model. However, our approach only identified phosphatases modulating the growth pathway but did not enable us to link phosphatases to specific substrates.

For this purpose, we set up to develop an experimental strategy that combines the functional information obtained with the siRNA screening and PPI network context information. We first enriched the literature derived interactome of six phosphatases and two phosphatase accessory subunits by affinity purification experiments of phosphatase complex followed by quantitative mass spectrometry based proteomics in cancer cells stimulated with TNFα. By this approach we were able to recapitulate most of the interactions occurring between the catalytic, scaffold, and regulatory subunit of the PP2A holoenzyme, confirming the robustness of our approach. The resulting interactome is completely connected, since each phosphatase shares at least one ligand with one of the remaining phosphatases. For instance the tyrosine phosphatase PTPN21 and the catalytic subunit of the serine threonine phosphatase PP2A share a common group of interactors, mainly involved in controlling cell metabolism. We observed that the phosphatase interactome is largely insensitive to stimulation with TNFα, suggesting that these interactions may be either constitutive or triggered by other types of stimuli. For instance, while the DUSP26-ATM interaction is not modulated by TNFα, we show that nutrients and amino acids deprivation increases the binding (Supplementary Material, Figure S1A), suggesting that these proteins may play a role in controlling the autophagy process. Indeed, we have previously shown that the siRNA interference of DUSP26 results in a decrease of the autophagy marker LC3, while much evidence suggest that the ATM kinase promotes the autophagy induced by ionizing radiation and ROS (Liang et al., 2013; Tripathi et al., 2013). In addition, as shown in Figure 3, about 50% of the PPP3CA interactions are negatively modulated by TNFα, including the binding to its activator subunit calmodulin. This result suggests that the TNFα stimulation may have an inhibitory role on PPP3CA activity. However, Fernandez et al. have recently demonstrated that in reactive, but not in quiescent astrocytes, PPP3CA dephosphorylates the transcription factor Foxo3 in response to TNFα, suggesting that depending on the cells type, this phosphatase may have opposite functions (Fernandez et al., 2012).

Interestingly our experimental approach enabled us to identify a novel interaction between the scaffold protein GRB2 and the tyrosine phosphatase PTPN21. Here, we report that PTPN21 binds the C-terminal SH3 domain of GRB2 *in vitro*, but it does not dephosphorylate its phosphotyrosine residues. Indeed our affinity purification experiment failed to identify known phosphatase substrates that had already been described in the literature. This observation is not surprising if we consider that phosphatases rapidly dephosphorylate the substrate and the phosphatase-substrate interaction is so transient and weak that coimmunoprecipitation-based approaches likely fail to identify phosphatase substrates. In addition, while most protein kinases recognize a specific amino acid motifs on their targets, phosphatase substrates specificity is weaker and mainly based on the interaction with regulatory subunits (Roy and Cyert, 2009).

To infer new phosphatase substrates, we have here outlined a combined experimental-bioinformatic strategy based on the integration of the phosphatase interactome with network context information, extracted from the *mentha* PPI database (Calderone et al., 2013). Although this approach lead us to recover some of the phosphatase-substrate relationships already described in literature, we are aware of some relations that are missed by our approach [e.g., the RAF1 dephosphorylation by PPP2CA (Dent et al., 1995)]. These failures can be explained by several factors: (1) some interactions may be cell type dependent or rely on specific stimulations; (2) some phosphatase partners may have very low level of expression that remains undetected in our affinity purification experiments and (3) some PPI relations may have not been reported yet or may have not been annotated in *mentha*. In addition we want to stress that we used rather stringent filtering criteria to reduce the total number of inferred phosphatase-scaffold-substrate complexes. This might increase the chance of missing already validated enzyme-substrate relationships or of identifying new interesting regulation mechanisms. If desirable, these criteria can be relaxed at the cost of increasing the noise of false positives.

In essence our method combines functional information with the interactome and analyses the resulting graph to identify paths between phosphatases and putative substrates. By this approach new substrates may be inferred or alternatively proteins that form molecular bridges between the phosphatase and the substrates can be identified. To assess the robustness and reliability of our strategy, two specific cases were analyzed. Firstly we demonstrated that DUSP18 induces SHP2 dephosphorylation. Our siRNA screening revealed that DUSP18 negatively controls ERK phosphorylation (Sacco et al., 2012a). This is consistent with SHP2 being a positive modulator of the MAPK signaling (Cai et al., 2002). Here we infer that catalase acts as a bridge to enable the DUSP18 mediated de-phosphorylation of SHP2. Although our approach does not identify the specific SHP2 tyrosine residues dephosphorylated by DUSP18, we demonstrated that the C-terminal residues involved in the GRB2 interaction are not targeted by the phosphatase (Figure 5). DUSP18 may negatively controls the MAPK signaling by dephosphorylating and inactivating SHP2. Finally, we demonstrated that SCRIB acts as a bridge to mediate the dephosphorylation of ERK by DUSP26 (Figure 7B). DUSP26 is a poorly characterized dual specificity phosphatase whose negative regulation of the MAPK signaling has been already reported.

Taken together these observations show that the combination of the topological information contained in the phosphatase interactome with functional information obtained by siRNA screening can be valid tool to infer new phosphatase substrates and modes of targeting.

## Materials and methods

### Antibodies and reagents

Anti-HA, anti-FLAG and anti-Flag M1 agarose beads and anti DUSP26 were from Sigma; anti-SHP2 and anti SCRIB were from Santa Cruz Biotechnology; anti-GRB2 and anti-4G10 was from Upstate Biotechnology, Inc. Peroxidase-conjugated anti-rabbit, anti-mouse and anti-goat secondary antibodies were from Jackson ImmunoResearch. PPP2CA, PTPN3, PTPN21, DUSP26, PPP2R3C encoding plasmids were purchased from OpenBiosystem. DUSP18, PPP2R1A, and PPP3CA constructs were kindly provided by Marc Vidal. Phosphatase cDNAs were cloned in pDNOR vector (Invitrogen) and cloned in the SF-TAP plasmid by using the Gateway Recominant Cloning Technology from Invitrogen. The cDNA of DUSP26 was also cloned in Pet28 and PC-DNA plasmids. HA-DLC1 was kindly provided by Prof. Cecconi. The cDNA encoding SRC Y527F was cloned in pSGT (Gonfloni et al., 1997). HA-SCRIB, PDZ3-GST, and PDZ4-GST were a generous gift of L. Banks. Construct containing human SCRIB PDZ1-2 and 1-4 (aa 728-1630) were cloned in pGex2TK.

### Cell culture

Cells were maintained in a humidified atmosphere at 37°C and 5% CO2 in Dulbecco's modified Eagle's medium (Invitrogen), supplemented with 10% fetal bovine serum (Sigma) and 0.1% penicillin/streptomycin (Invitrogen). For SILAC experiments, SILAC DMEM (PAA, Pasching, Austria) deficient of L-Lysine and L-Arginine, supplemented with 10% (v/v) dialyzed fetal bovine serum (FBS; PAA, Pasching, Austria), 50 units/ml Penicillin, 0.05 mg/ml Streptomycin and 0.55 mM lysine, 0.4 mM arginine was used. Light labeled medium was supplemented with ^12^C_6_, ^14^N_2_ lysine and ^12^C_6_, ^14^N_4_ arginine, medium labeled medium with 4.4.5.5-D_4_-L-Lysine and ^13^C_6_−^14^N_4_-L-Arginine and heavy labeled medium with ^13^C_6_^15^N_2_-L-Lysine and ^13^C_6_^15^N_4_-L-Arginine. Proline was added to a final concentration of 0.5 mM to prevent arginine to proline conversion (Bendall et al., 2008), which could impair the quantification. All amino acids were purchased from Silantes. Human epithelial carcinoma (HeLa) cells were purchased from the ATCC. HeLa cells were transfected with Lipofectamine 2000 (Invitrogen) according to manufacturer's protocol.

### Affinity purification of protein complexes

For one step Strep purifications, SF-TAP tagged proteins and associated protein complexes were purified essentially as described earlier (Gloeckner et al., 2007; Boldt et al., 2011). HeLa cells, transiently expressing the SF-TAP tagged constructs or SF-TAP alone as control were either stimulated with 50 ng/ml TNFα or mock treated. They were next lysed in lysis buffer (containing 150 mM NaCl, 50 mM Tris-HCl, 1% Nonidet P-40, and 0.25% sodium deoxycholate, protease inhibitor cocktail (Roche) and phosphatase inhibitor cocktails II and III (Sigma-Aldrich), for 20 min at 4°C. After sedimentation of nuclei at 10,000 × g for 10 min, the protein concentration of the lysates were determined by a Bradford assay before equal amounts of the cleared lysates were transferred to Strep-Tactin-Superflow beads (IBA) and incubated for 1 h before the resin was washed three times with wash buffer (TBS containing 0.1% NP-40, phosphatase inhibitor cocktail I and II). The protein complexes were eluted by incubation for 10 min in Strep-elution buffer (IBA). Following elution, the corresponding samples were combined. The combined samples were concentrated using 10 kDa cut-off VivaSpin 500 centrifugal devices (Sartorius Stedim Biotech) and pre-fractionated using SDS-Page and in-gel tryptic cleavage as described elsewhere (Gloeckner et al., 2009).

### Mass spectrometry and data analysis

LC-MS/MS analysis was performed on an Ultimate3000 nano RSLC system (Thermo Fisher Scientific) coupled to a LTQ Orbitrap Velos mass spectrometer (Thermo Fisher Scientific) by a nano spray ion source. Tryptic peptide mixtures were automatically injected and separated by a linear gradient from 5 to 40% of buffer B in buffer A (2% acetonitrile, 0.1% formic acid in HPLC grade water) in buffer A (0.1% formic acid in HPLC grade water) at a flow rate of 300 nl/min over 90 min. Remaining peptides were eluted by a short gradient from 40 to 100% buffer B in 5 min. The eluted peptides were analyzed by the LTQ Orbitrap Velos mass spectrometer. From the high resolution MS pre-scan with a mass range of 300–1500, the 10 most intense peptide ions were selected for fragment analysis in the linear ion trap if they exceeded an intensity of at least 500 counts and if they were at least doubly charged. The normalized collision energy for CID was set to a value of 35 and the resulting fragments were detected with normal resolution in the linear ion trap. The lock mass option was activated, the background signal with a mass of 445.12002 was used as lock mass (Olsen et al., 2005). Every ion selected for fragmentation, was excluded for 20 s by dynamic exclusion. For SILAC experiments, all acquired spectra were processed and analyzed using the MaxQuant software (Cox and Mann, 2008) (version 1.0.13.13) and the human specific IPI database version 3.52 (http://www.maxquant.org/) in combination with Mascot (Matrix Science, version 2.2). Cysteine carbamidomethylation was selected as fixed modification, methionine oxidation and protein acetylation were allowed as variable modifications. The peptide and protein false discovery rates were set to 1%. Contaminants like keratins were removed. Proteins, identified and quantified by at least two unique peptides were considered for further analysis. The significance values were determined by Perseus tool using significance B. Those proteins whose ratio was greater than 1.9 and significance B was lesser than 0.1 were considered significantly enriched.

### Pull-down assay

After 24 h of transfection, confluent HeLa cells were washed with ice-cold PBS and lysed in RIPA buffer (150 mm NaCl, 50 mm Tris-HCl, 1% Nonidet P-40, 0.25% sodium deoxycholate) supplemented with 1 mm pervanadate, 1 mm NaF, protease inhibitor mixture 200× (Sigma), inhibitor phosphatase mixture I and II 100× (Sigma). The samples were kept on ice for 30 min and centrifuged at 15,000 rpm at 4°C for 30 min. The supernatant was collected, and the total amount of protein was determined by Bradford colorimetric assay (Bio-Rad). The whole cell lysates were incubated with 50 μg of the indicated GST fusion protein at 4°C for 1 h. Thus, glutathione-Sepharose 4B beads were blocked by incubating with 3% bovine serum albumin with rocking at 4°C for 1 h, and then after centrifugation for 3 min at 4000 × g, at 4°C, the dry beads were bound to lysates mixed with GST fusion proteins at 4°C for 1 h. The supernatant was discarded by centrifugation, and the beads were washed six times with lysis buffer for 3 min at 4000 × g, at 4°C, and then the dry beads were resuspended in SDS sample buffer, boiled and analyzed by SDS-PAGE and Western blotting on nitrocellulose membrane.

### Immunoprecipitation and immunoblot analysis

HeLa cells were lysed as described previously. The whole cell lysates were incubated with anti-Flag antibody conjugated to Sepharose beads over-night at 4°C. The beads were washed with lysis buffer, and the immunoprecipitated proteins were separated by SDS-PAGE, transferred onto a nitrocellulose membrane, and immunoblotted with antibodies. The immunoreactions were visualized using ECL detection system (Amersham Biosciences).

### Conflict of interest statement

The authors declare that the research was conducted in the absence of any commercial or financial relationships that could be construed as a potential conflict of interest.

## Supplementary material

Figure S1 **Validation of some of the newly identified phosphatase interactions (A) HeLa cells were transiently transfected with Flag-DUSP26 expression plasmid**. Twenty-four hours post transfection, cells were serum and amino acids starved for 1 h or left untreated and then lysed. Whole protein extracts were immunoprecipitated with anti-Flag antibody to purify the DUSP26 phosphatase. The membranes were probed with anti-ATM (WB: α-ATM) and anti-Flag (WB: α-Flag) antibodies. **(B)** HeLa cells were transiently co-transfected with Flag-PTPN21 and with HA-DLC1 expression plasmids. Twenty-four hours post transfection, cells were serum and amino acids starved for 1 h or left untreated and then lysed. Whole protein extracts were immunoprecipitated with anti-Flag antibody to purify PTPN21 phosphatase. The membranes were probed with anti-HA (WB: α-HA), anti-Flag (WB: α-Flag) and anti-GRB2 antibodies. **(C)** HeLa cells were transiently co-transfected with Flag-PPP2CA and with HA-DLC1 expression plasmids. Twenty-four hours post transfection, cells were serum and amino acids starved for 1 h or left untreated and then lysed. Whole protein extracts were immunoprecipitated with anti-Flag antibody to purify PTPN21 phosphatase. The membranes were probed with anti-HA (WB: α-HA) and anti-Flag (WB: α-Flag) antibodies.

Table S1 **Protein groups identified by mass spectrometry based proteomics of phosphatase pull-down are reported with protein quantification, number of peptides and intensities**.

Table S2 **After statistical analysis, for each phosphatase the corresponding interactor is reported**. In the “Phosph-SF” column, the intensity value of each interactor in phosphatase transfected cells was divided by its intensity in not transfected cells (Control). In the “PhosphTNF-Phosph” column, the intensity value of each interactor in cells over-expressing the phosphatase and stimulated with TNFα was divided by its intensity in transfected unstimulated cells. Finally in “PhosphTNF-SF” column, the intensity value of each interactor in cells over-expressing the phosphatase and stimulated with TNFα was divided by its intensity in not transfected cells. For each ratio, the corresponding significance B is reported.

Table S3 **List of common contaminants was collected from the literature**.

Table S4 **Experimental data describing the functional relationships between signaling proteins in the pathways of interest were collected from the literature (PMID column)**. Each enzyme-substrate relationship is described as activating (1) or inhibitory (-1). For each protein, Uniprot ID and gene name have been reported.

Table S5 **Experimental and literature extracted binary interactions, describing the paths from a phosphatase to its target in the RAS-PI3K pathway**.

## References

[B1] Bardelli A. Velculescu V. E. (2005). Mutational analysis of gene families in human cancer. Curr. Opin. Genet. Dev. 15, 5–12 10.1016/j.gde.2004.12.009 15661527

[B2] Bendall S. C. Hughes C. Stewart M. H. Doble B. Bhatia M. Lajoie G. A. (2008). Prevention of amino acid conversion in SILAC experiments with embryonic stem cells. Mol. Cell. Proteomics 7, 1587–1597 10.1074/mcp.M800113-MCP200 18487603 PMC2556023

[B3] Blanchetot C. Chagnon M. Dube N. Halle M. Tremblay M. L. (2005). Substrate-trapping techniques in the identification of cellular PTP targets. Methods 35, 44–53 10.1016/j.ymeth.2004.07.007 15588985

[B4] Boldt K. Mans D. A. Won J. Van Reeuwijk J. Vogt A. Kinkl N. (2011). Disruption of intraflagellar protein transport in photoreceptor cilia causes Leber congenital amaurosis in humans and mice. J. Clin. Invest. 121, 2169–2180 10.1172/JCI45627 21606596 PMC3104757

[B5] Breitkreutz A. Choi H. Sharom J. R. Boucher L. Neduva V. Larsen B. (2010). A global protein kinase and phosphatase interaction network in yeast. Science 328, 1043–1046 10.1126/science.1176495 20489023 PMC3983991

[B6] Brill L. M. Xiong W. Lee K. B. Ficarro S. B. Crain A. Xu Y. (2009). Phosphoproteomic analysis of human embryonic stem cells. Cell Stem Cell 5, 204–213 10.1016/j.stem.2009.06.002 19664994 PMC2726933

[B7] Cai T. Nishida K. Hirano T. Khavari P. A. (2002). Gab1 and SHP-2 promote Ras/MAPK regulation of epidermal growth and differentiation. J. Cell Biol. 159, 103–112 10.1083/jcb.200205017 12370245 PMC2173502

[B8] Calderone A. Castagnoli L. Cesareni G. (2013). mentha: a resource for browsing integrated protein-interaction networks. Nat. Methods 10, 690–691 10.1038/nmeth.2561 23900247

[B9] Cardone L. Carlucci A. Affaitati A. Livigni A. Decristofaro T. Garbi C. (2004). Mitochondrial AKAP121 binds and targets protein tyrosine phosphatase D1, a novel positive regulator of src signaling. Mol. Cell Biol. 24, 4613–4626 10.1128/MCB.24.11.4613-4626.2004 15143158 PMC416429

[B10] Carducci M. Perfetto L. Briganti L. Paoluzi S. Costa S. Zerweck J. (2012). The protein interaction network mediated by human SH3 domains. Biotechnol. Adv. 30, 4–15 10.1016/j.biotechadv.2011.06.012 21740962

[B11] Carlucci A. Porpora M. Garbi C. Galgani M. Santoriello M. Mascolo M. (2010). PTPD1 supports receptor stability and mitogenic signaling in bladder cancer cells. J. Biol. Chem. 285, 39260–39270 10.1074/jbc.M110.174706 20923765 PMC2998146

[B12] Couzens A. L. Knight J. D. Kean M. J. Teo G. Weiss A. Dunham W. H. (2013). Protein interaction network of the mammalian Hippo pathway reveals mechanisms of kinase-phosphatase interactions. Sci. Signal. 6, rs15 10.1126/scisignal.2004712 24255178

[B13] Cox J. Mann M. (2008). MaxQuant enables high peptide identification rates, individualized p.p.b.-range mass accuracies and proteome-wide protein quantification. Nat. Biotechnol. 26, 1367–1372 10.1038/nbt.1511 19029910

[B14] Dent P. Jelinek T. Morrison D. K. Weber M. J. Sturgill T. W. (1995). Reversal of Raf-1 activation by purified and membrane-associated protein phosphatases. Science 268, 1902–1906 10.1126/science.7604263 7604263

[B15] Duan L. Cobb M. H. (2010). Calcineurin increases glucose activation of ERK1/2 by reversing negative feedback. Proc. Natl. Acad. Sci. U.S.A. 107, 22314–22319 10.1073/pnas.1016630108 21135229 PMC3009760

[B16] Fernandez A. M. Jimenez S. Mecha M. Davila D. Guaza C. Vitorica J. (2012). Regulation of the phosphatase calcineurin by insulin-like growth factor I unveils a key role of astrocytes in Alzheimer's pathology. Mol. Psychiatry 17, 705–718 10.1038/mp.2011.128 22005929

[B16a] Gonfloni S. Williams J. C. Hattula K. Weijland A. Wierenga R. K. Superti-Furga G. (1997). The role of the linker between the SH2 domain and catalytic domain in the regulation and function of Src. EMBO J. 16, 7261–7271 10.1093/emboj/16.24.7261 9405355 PMC1170326

[B17] Gloeckner C. J. Boldt K. Schumacher A. Roepman R. Ueffing M. (2007). A novel tandem affinity purification strategy for the efficient isolation and characterisation of native protein complexes. Proteomics 7, 4228–4234 10.1002/pmic.200700038 17979178

[B18] Gloeckner C. J. Boldt K. Ueffing M. (2009). Strep/FLAG tandem affinity purification (SF-TAP) to study protein interactions. Curr. Protoc. Protein Sci. Chapter 19:Unit19.20. 10.1002/0471140864.ps1920s57 19688738

[B19] Goudreault M. D'Ambrosio L. M. Kean M. J. Mullin M. J. Larsen B. G. Sanchez A. (2009). A PP2A phosphatase high density interaction network identifies a novel striatin-interacting phosphatase and kinase complex linked to the cerebral cavernous malformation 3 (CCM3) protein. Mol. Cell. Proteomics 8, 157–171 10.1074/mcp.M800266-MCP200 18782753 PMC2621004

[B20] Graves J. D. Krebs E. G. (1999). Protein phosphorylation and signal transduction. Pharmacol. Ther. 82, 111–121 10.1016/S0163-7258(98)00056-4 10454190

[B21] Guo A. Villen J. Kornhauser J. Lee K. A. Stokes M. P. Rikova K. (2008). Signaling networks assembled by oncogenic EGFR and c-Met. Proc. Natl. Acad. Sci. U.S.A. 105, 692–697 10.1073/pnas.0707270105 18180459 PMC2206598

[B22] Hahn K. Miranda M. Francis V. A. Vendrell J. Zorzano A. Teleman A. A. (2010). PP2A regulatory subunit PP2A-B' counteracts S6K phosphorylation. Cell Metab. 11, 438–444 10.1016/j.cmet.2010.03.015 20444422

[B23] Han S. Williams S. Mustelin T. (2000). Cytoskeletal protein tyrosine phosphatase PTPH1 reduces T cell antigen receptor signaling. Eur. J. Immunol. 30, 1318–1325 10.1002/(SICI)1521-4141(200005)30:5%3C1318::AID-IMMU1318%3E3.0.CO;2-G 10820377

[B24] He H. Liu X. Lv L. Liang H. Leng B. Zhao D. (2014). Calcineurin suppresses AMPK-dependent cytoprotective autophagy in cardiomyocytes under oxidative stress. Cell Death Dis. 5, e997 10.1038/cddis.2013.533 24434520 PMC4040710

[B25] Hornbeck P. V. Kornhauser J. M. Tkachev S. Zhang B. Skrzypek E. Murray B. (2012). PhosphoSitePlus: a comprehensive resource for investigating the structure and function of experimentally determined post-translational modifications in man and mouse. Nucleic Acids Res. 40, D261–D270 10.1093/nar/gkr1122 22135298 PMC3245126

[B26] Hu Y. Mivechi N. F. (2006). Association and regulation of heat shock transcription factor 4b with both extracellular signal-regulated kinase mitogen-activated protein kinase and dual-specificity tyrosine phosphatase DUSP26. Mol. Cell. Biol. 26, 3282–3294 10.1128/MCB.26.8.3282-3294.2006 16581800 PMC1446944

[B27] Huang da W. Sherman B. T. Lempicki R. A. (2009). Systematic and integrative analysis of large gene lists using DAVID bioinformatics resources. Nat. Protoc. 4, 44–57 10.1038/nprot.2008.211 19131956

[B28] Hurov K. E. Cotta-Ramusino C. Elledge S. J. (2010). A genetic screen identifies the Triple T complex required for DNA damage signaling and ATM and ATR stability. Genes Dev. 24, 1939–1950 10.1101/gad.1934210 20810650 PMC2932975

[B29] Julien S. G. Dube N. Hardy S. Tremblay M. L. (2011). Inside the human cancer tyrosine phosphatome. Nat. Rev. Cancer 11, 35–49 10.1038/nrc2980 21179176

[B30] Kao S. C. Wu H. Xie J. Chang C. P. Ranish J. A. Graef I. A. (2009). Calcineurin/NFAT signaling is required for neuregulin-regulated Schwann cell differentiation. Science 323, 651–654 10.1126/science.1166562 19179536 PMC2790385

[B31] Kim H. Lee H. J. Oh Y. Choi S. G. Hong S. H. Kim H. J. (2014). The DUSP26 phosphatase activator adenylate kinase 2 regulates FADD phosphorylation and cell growth. Nat. Commun. 5, 3351 10.1038/ncomms4351 24548998 PMC3948464

[B32] Li X. Wilmanns M. Thornton J. Kohn M. (2013). Elucidating human phosphatase-substrate networks. Sci. Signal. 6, rs10 10.1126/scisignal.2003203 23674824

[B33] Liang N. Jia L. Liu Y. Liang B. Kong D. Yan M. (2013). ATM pathway is essential for ionizing radiation-induced autophagy. Cell. Signal. 25, 2530–2539 10.1016/j.cellsig.2013.08.010 23993957

[B34] Liberti S. Sacco F. Calderone A. Perfetto L. Iannuccelli M. Panni S. (2013). HuPho: the human phosphatase portal. FEBS J. 280, 379–387 10.1111/j.1742-4658.2012.08712.x 22804825

[B35] Magnaudeix A. Wilson C. M. Page G. Bauvy C. Codogno P. Leveque P. (2013). PP2A blockade inhibits autophagy and causes intraneuronal accumulation of ubiquitinated proteins. Neurobiol. Aging 34, 770–790 10.1016/j.neurobiolaging.2012.06.026 22892312

[B36] Mann M. Jensen O. N. (2003). Proteomic analysis of post-translational modifications. Nat. Biotechnol. 21, 255–261 10.1038/nbt0303-255 12610572

[B37] Manning G. Plowman G. D. Hunter T. Sudarsanam S. (2002a). Evolution of protein kinase signaling from yeast to man. Trends Biochem. Sci. 27, 514–520 10.1016/S0968-0004(02)02179-5 12368087

[B38] Manning G. Whyte D. B. Martinez R. Hunter T. Sudarsanam S. (2002b). The protein kinase complement of the human genome. Science 298, 1912–1934 10.1126/science.1075762 12471243

[B39] Meixner A. Boldt K. Van Troys M. Askenazi M. Gloeckner C. J. Bauer M. (2011). A QUICK screen for Lrrk2 interaction partners–leucine-rich repeat kinase 2 is involved in actin cytoskeleton dynamics. Mol. Cell. Proteomics 10, M110 001172 10.1074/mcp.M110.001172 20876399 PMC3013447

[B40] Nagasaka K. Pim D. Massimi P. Thomas M. Tomaic V. Subbaiah V. K. (2010). The cell polarity regulator hScrib controls ERK activation through a KIM site-dependent interaction. Oncogene 29, 5311–5321 10.1038/onc.2010.265 20622900

[B41] Nagasaka K. Seiki T. Yamashita A. Massimi P. Subbaiah V. K. Thomas M. (2013). A novel interaction between hScrib and PP1gamma downregulates ERK signaling and suppresses oncogene-induced cell transformation. PLoS ONE 8:e53752 10.1371/journal.pone.0053752 23359326 PMC3554735

[B42] Olsen J. V. de Godoy L. M. Li G. Macek B. Mortensen P. Pesch R. (2005). Parts per million mass accuracy on an Orbitrap mass spectrometer via lock mass injection into a C-trap. Mol. Cell. Proteomics 4, 2010–2021 10.1074/mcp.T500030-MCP200 16249172

[B43] Ong S. E. Blagoev B. Kratchmarova I. Kristensen D. B. Steen H. Pandey A. (2002). Stable isotope labeling by amino acids in cell culture, SILAC, as a simple and accurate approach to expression proteomics. Mol. Cell. Proteomics 1, 376–386 10.1074/mcp.M200025-MCP200 12118079

[B44] Patterson K. I. Brummer T. Daly R. J. O'Brien P. M. (2010). DUSP26 negatively affects the proliferation of epithelial cells, an effect not mediated by dephosphorylation of MAPKs. Biochim. Biophys. Acta 1803, 1003–1012 10.1016/j.bbamcr.2010.03.014 20347885

[B45] Rikova K. Guo A. Zeng Q. Possemato A. Yu J. Haack H. (2007). Global survey of phosphotyrosine signaling identifies oncogenic kinases in lung cancer. Cell 131, 1190–1203 10.1016/j.cell.2007.11.025 18083107

[B46] Roy J. Cyert M. S. (2009). Cracking the phosphatase code: docking interactions determine substrate specificity. Sci. Signal. 2, re9 10.1126/scisignal.2100re9 19996458

[B47] Sacco F. Gherardini P. F. Paoluzi S. Saez-Rodriguez J. Helmer-Citterich M. Ragnini-Wilson A. (2012a). Mapping the human phosphatome on growth pathways. Mol. Syst. Biol. 8, 603 10.1038/msb.2012.36 22893001 PMC3435503

[B48] Sacco F. Perfetto L. Castagnoli L. Cesareni G. (2012b). The human phosphatase interactome: an intricate family portrait. FEBS Lett. 586, 2732–2739 10.1016/j.febslet.2012.05.008 22626554 PMC3437441

[B49] Shang X. Vasudevan S. A. Yu Y. Ge N. Ludwig A. D. Wesson C. L. (2010). Dual-specificity phosphatase 26 is a novel p53 phosphatase and inhibits p53 tumor suppressor functions in human neuroblastoma. Oncogene 29, 4938–4946 10.1038/onc.2010.244 20562916 PMC7580258

[B50] Skarra D. V. Goudreault M. Choi H. Mullin M. Nesvizhskii A. I. Gingras A. C. (2011). Label-free quantitative proteomics and SAINT analysis enable interactome mapping for the human Ser/Thr protein phosphatase 5. Proteomics 11, 1508–1516 10.1002/pmic.201000770 21360678 PMC3086140

[B51] Taniguchi T. Garcia-Higuera I. Xu B. Andreassen P. R. Gregory R. C. Kim S. T. (2002). Convergence of the fanconi anemia and ataxia telangiectasia signaling pathways. Cell 109, 459–472 10.1016/S0092-8674(02)00747-X 12086603

[B52] Tanuma N. Nomura M. Ikeda M. Kasugai I. Tsubaki Y. Takagaki K. (2009). Protein phosphatase Dusp26 associates with KIF3 motor and promotes N-cadherin-mediated cell-cell adhesion. Oncogene 28, 752–761 10.1038/onc.2008.431 19043453

[B53] Tonikian R. Zhang Y. Sazinsky S. L. Currell B. Yeh J. H. Reva B. (2008). A specificity map for the PDZ domain family. PLoS Biol. 6:e239 10.1371/journal.pbio.0060239 18828675 PMC2553845

[B54] Tonks N. K. (2006). Protein tyrosine phosphatases: from genes, to function, to disease. Nat. Rev. Mol. Cell Biol. 7, 833–846 10.1038/nrm2039 17057753

[B55] Tremblay M. L. (2009). The PTP family photo album. Cell 136, 213–214 10.1016/j.cell.2009.01.006 19167325

[B56] Tripathi D. N. Chowdhury R. Trudel L. J. Tee A. R. Slack R. S. Walker C. L. (2013). Reactive nitrogen species regulate autophagy through ATM-AMPK-TSC2-mediated suppression of mTORC1. Proc. Natl. Acad. Sci. U.S.A. 110, E2950–E2957 10.1073/pnas.1307736110 23878245 PMC3740898

[B57] Wera S. Hemmings B. A. (1995). Serine/threonine protein phosphatases. Biochem. J. 311(pt 1), 17–29 7575450 10.1042/bj3110017 PMC1136113

[B58] Yano S. Arroyo N. Yano N. (2004). SHP2 binds catalase and acquires a hydrogen peroxide-resistant phosphatase activity via integrin-signaling. FEBS Lett. 577, 327–332 10.1016/j.febslet.2004.10.011 15556604

