# Supplementary figures and images for: Combining affinity proteomics and network context to identify new phosphatase substrates and adapters in growth pathways
Source: Front Genet. 2014 May 7;5:115. doi: 10.3389/fgene.2014.00115 (PMC4019850; doi:10.3389/fgene.2014.00115)

A

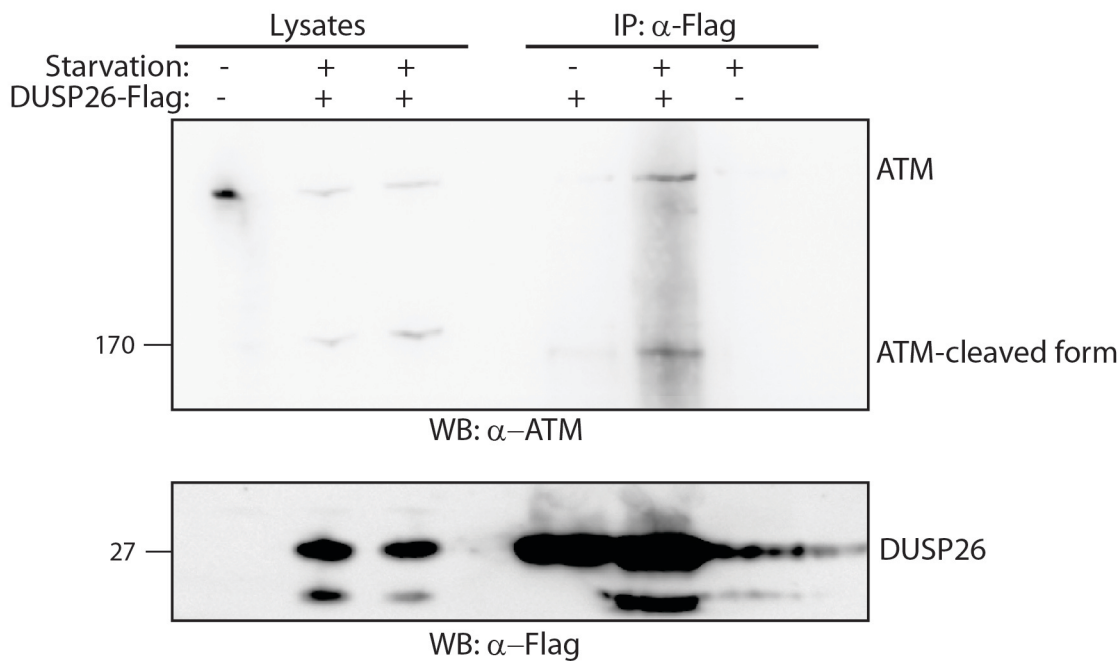

B

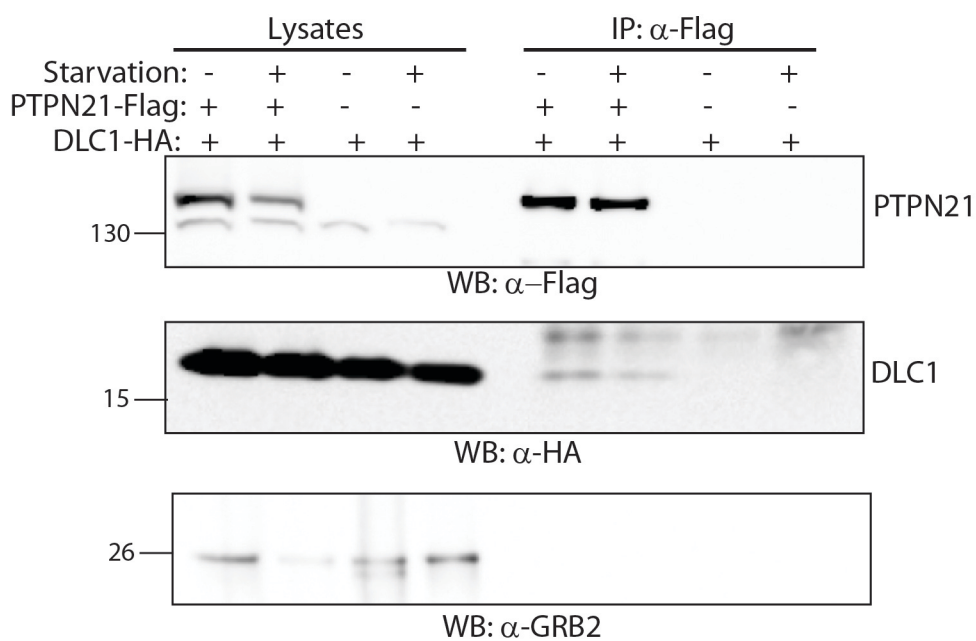

C

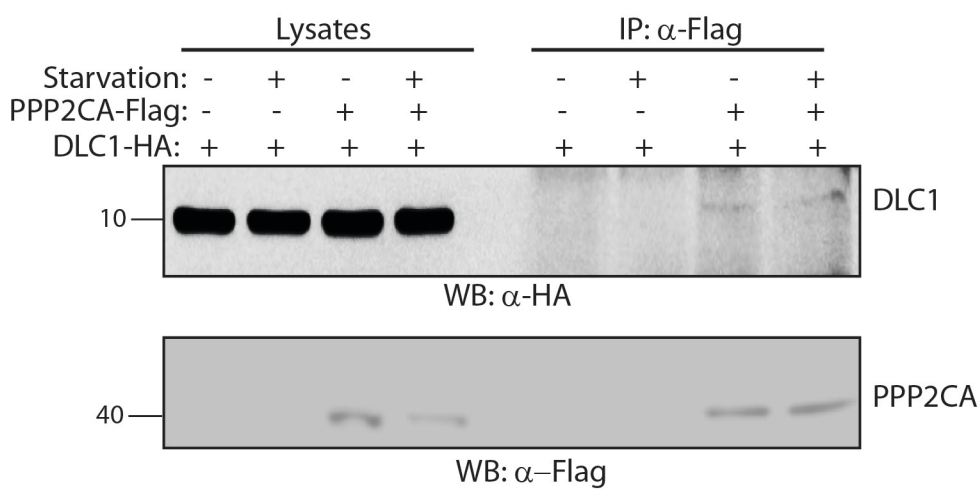

Supplement: Figure S1 — Validation of some of the newly identified phosphatase interactions (A) HeLa cells were transiently transfected with Flag-DUSP26 expression plasmid. Twenty-four hours post transfection, cells were serum and amino acids starved for 1 h or left untreated and then lysed. Whole protein extracts were immunoprecipitated with anti-Flag antibody to purify the DUSP26 phosphatase. The membranes were probed with anti-ATM (WB: α-ATM) and anti-Flag (WB: α-Flag) antibodies. (B) HeLa cells were transiently co-transfected with Flag-PTPN21 and with HA-DLC1 expression plasmids. Twenty-four hours post transfection, cells were serum and amino acids starved for 1 h or left untreated and then lysed. Whole protein extracts were immunoprecipitated with anti-Flag antibody to purify PTPN21 phosphatase. The membranes were probed with anti-HA (WB: α-HA), anti-Flag (WB: α-Flag) and anti-GRB2 antibodies. (C) HeLa cells were transiently co-transfected with Flag-PPP2CA and with HA-DLC1 expression plasmids. Twenty-four hours post transfection, cells were serum and amino acids starved for 1 h or left untreated and then lysed. Whole protein extracts were immunoprecipitated with anti-Flag antibody to purify PTPN21 phosphatase. The membranes were probed with anti-HA (WB: α-HA) and anti-Flag (WB: α-Flag) antibodies. [file Presentation1.PDF]
